# Efficacy of Liposomal Bupivacaine in Regional Nerve Blocks for the Below-Knee Amputation: A Retrospective Cohort Study

**DOI:** 10.7759/cureus.112613

**Published:** 2026-07-13

**Authors:** Sai Mandalapu, Natasha Miner, Abhi Somenhalli, Malte Krapp, Bright Hyun Woo Kim, Anna Ng-Pellegrino

**Affiliations:** 1 General Surgery, St. Luke's University Health Network, Bethlehem, USA; 2 Lewis Katz School of Medicine, Temple University, Philadelphia, USA; 3 Research and Innovations, St. Luke's University Health Network, Bethlehem, USA; 4 Anesthesiology and Perioperative Medicine, St. Luke's University Health Network, Bethlehem, USA

**Keywords:** below-knee amputation, liposomal bupivacaine, opioids, oral morphine equivalent, pain score, phantom-limb pain, postoperative pain, ultrasound guided regional nerve block

## Abstract

Introduction: Below-knee amputation (BKA) patients often struggle with postoperative pain as well as phantom limb pain. As excessive postoperative opioid use increases the risk of dependence, hyperalgesia, and decreased quality of life, regional nerve blocks using liposomal bupivacaine (LB) offer the promise of providing postoperative pain control while minimizing the need for opioids. This study aims to evaluate whether the use of LB in regional nerve blocks for BKA surgery prolongs postoperative analgesia compared with conventional local anesthetic blocks and no nerve blocks.

Methods: A retrospective cohort study was conducted on patients who underwent a BKA at a large community hospital system from 2019 to 2024, placing patients into three arms: ultrasound-guided nerve block with LB, ultrasound-guided nerve block with local anesthetic only, and no nerve block. The primary outcome was daily average oral morphine equivalent (OME) use within the first 48 hours postoperatively. The secondary outcome was average pain scores in the first 48 hours postoperatively.

Results: The use of LB in nerve blocks for BKA surgery led to a statistically significant difference in pain scores when compared with the other two groups.

Discussion: The much higher cost of LB compared to local anesthetic becomes a consideration in the context of less pain but similar opioid use.

## Introduction

Approximately 150,000 patients undergo lower extremity amputations in the US each year, with approximately 94% of amputations occurring due to diabetes and vascular disease [1,2]. With the rising frequency of these conditions, the number of amputation procedures is expected to increase over the next several decades, including below-knee amputations (BKAs) [2-5]. Severe postoperative pain remains an issue after a BKA procedure, which is compounded by a 50%-80% risk of developing phantom limb pain [6-9]. Severe postoperative pain is a consistent risk factor for developing chronic post-amputation pain, defined as pain lasting more than three months [10-12].

The foundation of medical management for BKAs involves multimodal pain analgesia, with opioids as needed [13]. However, opioids have undesirable side effects, including nausea/emesis, constipation, oversedation, respiratory depression, and increased hospital length of stay [14-18]. In addition, opioid-naïve patients undergoing major amputation procedures have a 20% risk of developing new persistent opioid use (NPOU), defined as filling an opioid prescription more than three months postoperatively [19-21]. As a result, utilizing regional analgesia has become paramount to optimizing an opioid-sparing strategy for BKA surgery and improving the quality of recovery for these patients.

The field of regional anesthesia has grown in the past two decades to provide new techniques for pain control after surgery [13,22-24]. When appropriate, regional anesthesia has been shown to reduce postoperative pain and postoperative delirium [25-27]. In 2011, the FDA approved the use of liposomal bupivacaine (LB) in shoulder arthroplasty procedures, which has been shown to extend the analgesic duration of nerve blocks up to 72 hours compared to nerve blocks with local anesthetic only [28,29]. However, recent meta-analyses have shown that LB is not universally superior to local anesthetic and instead depends on many factors, including the location on the body, injection technique, and the timeframe being studied [30-32]. Knowing the context in which LB is more effective becomes important when considering costs, as an equivalent dose of LB has been reported to range from 12 to 64 times more expensive than regular bupivacaine, depending on institutional costs [28,33]. In 2023, the FDA approved the use of LB in popliteal sciatic and adductor canal nerve blocks in adults [34]. Though it has been shown that regional nerve blocks with LB improve postoperative analgesia and reduce opioid use compared to no regional block [35], to our knowledge, there has been no study showing that LB is superior to an intermediate arm (regional nerve block with local anesthetic only) for BKA patients. 

This study investigates the hypothesis that regional nerve blocks with LB reduce pain and opioid consumption in the first 48 hours postoperatively, compared with regional blocks without LB and no regional block. Although this study analyzes acute pain and early opioid use, further investigation on this topic would involve the use of LB in BKA surgery and its impact on the risk of chronic post-amputation pain, phantom limb pain, and NPOU.

## Materials and methods

Institutional review board approval was obtained. A retrospective chart review was performed to identify all patients within two campuses of a large community hospital system who had undergone a BKA procedure from January 2019 to May 2024. The three arms of the study were defined by whether the patient received, preoperatively on the day of the BKA procedure: Group 1 - ultrasound-guided nerve block with LB; Group 2 - ultrasound-guided nerve block with local anesthetic (bupivacaine) only; and Group 3 - no nerve block. For the LB group, popliteal and femoral blocks were performed, each consisting of 10 cc of Exparel and 10 cc of 0.25% bupivacaine. Patients in the local-only group received 20 cc of 0.25% bupivacaine for both femoral and popliteal blocks. All blocks were performed by an anesthesia attending.

The primary outcome was daily average oral morphine equivalent (OME) use within the first 48 hours postoperatively. The secondary outcome was average pain scores (on a numerical rating scale of 0-10, with 0 being no pain and 10 being the worst pain imaginable) in the first 48 hours postoperatively. Pain scores were extracted for each patient using their average patient-reported 1-10 pain score obtained by nursing every four hours over the first two days postoperatively. OME was calculated using the Morphine Milligram Equivalent (MME) conversion table [35] to calculate the daily average OMEs taken by each patient.

**Figure 1:**
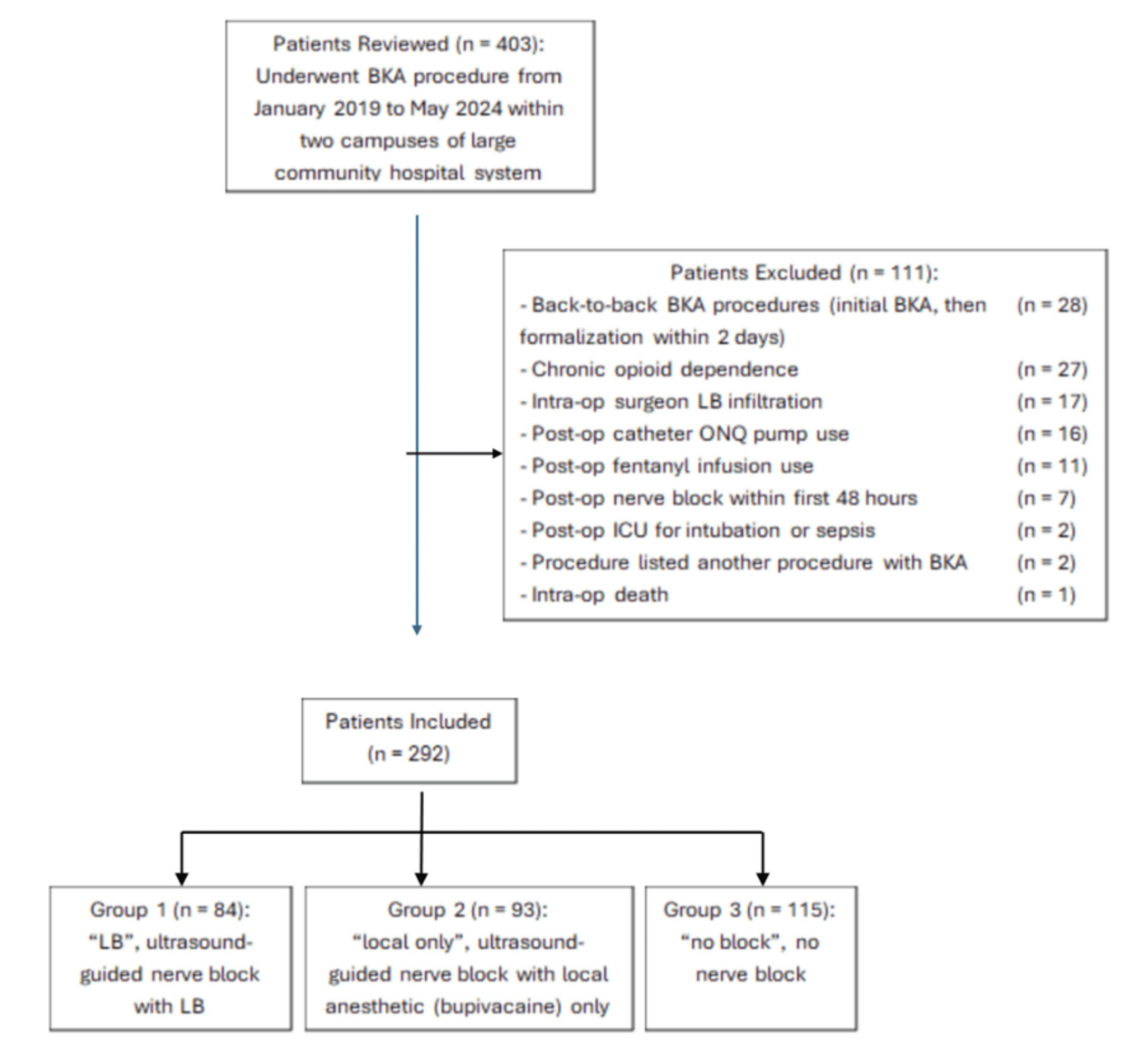
Flowchart of Patient Charts Reviewed, Excluded, and Included LB: liposomal bupivacaine; BKA: below-knee amputation

Patients were excluded if they received regional nerve blocks prior to the day of surgery, received intraoperative infiltration of their BKA stump by the surgeon with LB or local anesthetic, had another procedure performed along with the BKA, were pronounced deceased during the procedure, received additional regional nerve blocks during the first 48 hours postoperatively, had a postoperative catheter pain pump or fentanyl infusion, were transferred postoperatively to the ICU due to intubation or sepsis, had back-to-back BKA procedures (i.e. an initial BKA followed by formalization within two days), had chronic opioid dependence, were unable to consent, or were allergic to local anesthetic (Figure 1). 

For data safety, all study data were kept only on encrypted, password-protected computers. Initial patient data were not de-identified due to the need to incorporate patient demographics and operative records. However, after all data were collected, patient identifiers were deleted so that all information was de-identified.

For power analysis, as the primary outcome was OME use, it was predicted that the control cohort ("no block") would have a daily average OME use of 60, the intermediate cohort ("local only" regional nerve block) would have a daily average OME use of 50, and the "LB" regional nerve block cohort would have a daily average OME use of 40. To detect a difference of 33.3% (from 60 to 50 to 40), assuming a heterogeneous study population with a subsequent larger standard deviation (to provide the most conservative sample size estimate), at least 54 patients were deemed necessary for each arm (α = 0.05 and β = 0.20).

Statistical analysis of the final data was conducted using ANOVA and then two-tailed Student's t-tests to test for statistical significance between the three arms. For demographic data, ANOVA or chi-squared tests were conducted depending on the specific variables. Continuous variables were tested for normality with a Shapiro-Wilk test. Pairwise comparisons were performed using Tukey's Honestly Significant Difference test to adjust for multiple comparisons.

## Results

Of the total, 403 patients were identified as having undergone a BKA procedure from January 2019 to May 2024. These charts were reviewed, and 111 were excluded, with a full breakdown of the reasons shown in Figure 1. Thus, a total of 292 patients were included and analyzed in the study. Eighty-four patients were in Group 1 ("LB"), 93 were in Group 2 ("local only"), and 115 were in Group 3 ("no block"). A detailed breakdown of patient demographics can be found in Table 1. There was no statistically significant difference among the three arms in terms of age, gender, American Society of Anesthesiologists (ASA) score, body mass index (BMI), ketamine or dexmedetomidine use, hyperlipidaemia, heart failure, sleep apnea, cerebrovascular accident, anemia, hypothyroidism, smoking, or cancer. There were statistically significant differences, although none appeared to be clinically significant, among the three arms in elective cases (Group 3 had more), hypertension (Group 3 had more), coronary artery disease (Group 2 had more), deep vein thrombosis/pulmonary embolism (Group 1 had more), diabetes (Group 1 had less), gastroesophageal reflux disease (Group 1 had less), chronic kidney disease (Group 1 had less), and anxiety/depression (all three groups varied), as detailed in Table 1. 

**Table 1:** Patient Demographics Degrees of freedom for all chi-square tests: 2. ASA: American Society of Anesthesiologists; BMI: body mass index; HTN: hypertension; HLD: hyperlipidemia; CAD: coronary artery disease; CHF: congestive heart failure; DVT/PE: deep vein thrombosis/pulmonary embolism; OSA: obstructive sleep apnea; COPD: chronic obstructive pulmonary disease; DM: diabetes mellitus; CVA: cerebrovascular accident; GERD: gastroesophageal reflux disease; CKD: chronic kidney disease

Demographic	Ultrasound-Guided Nerve Block With LB (n = 84)	Ultrasound-Guided Nerve Block With Bupivacaine Only (n = 93)	No Nerve Block (n = 115)	Difference (p-value, effect size)
Age in years, mean (SD)	65.49 (10.89)	65.61 (11.77)	62.99 (11.79)	0.18
Male gender (%)	73.81	68.82	70.43	0.76, 0.03
ASA score, mean (SD)	3.36 (0.51)	3.39 (0.59)	3.35 (0.59)	0.88
BMI, mean (SD)	30.38 (7.93)	28.38 (6.19)	30.14 (8.73)	0.16
Ketamine or dexmedetomidine use (%)	16.67	27.96	26.09	0.17, 0.02
Elective case (%)	5.95	6.45	25.22	<0.01, <0.01
HTN (%)	89.29	91.40	100.00	<0.01, <0.01
HLD (%)	79.76	86.02	85.22	0.47, 0.03
CAD (%)	44.05	64.52	46.96	0.01, <0.01
CHF (%)	47.62	58.06	49.57	0.32, 0.02
DVT/PE (%)	16.67	8.60	5.22	0.02, <0.01
OSA (%)	21.43	33.33	26.96	0.21, 0.02
COPD (%)	15.48	23.66	22.61	0.35, 0.02
DM (%)	80.95	92.47	91.30	0.03, <0.01
CVA (%)	22.62	25.81	25.22	0.87, 0.04
GERD (%)	36.90	54.84	50.43	0.05, <0.01
CKD (%)	64.29	83.87	81.74	<0.01, <0.01
Anemia (%)	70.24	78.49	82.61	0.11, 0.02
Hypothyroidism (%)	22.62	29.03	21.74	0.43, 0.03
Smoking (%)	19.05	15.05	17.39	0.78, 0.04
Cancer (%)	10.71	22.58	15.65	0.10, 0.01
Anxiety/Depression (%)	39.29	60.22	51.30	0.02, <0.01

**Figure 2:**
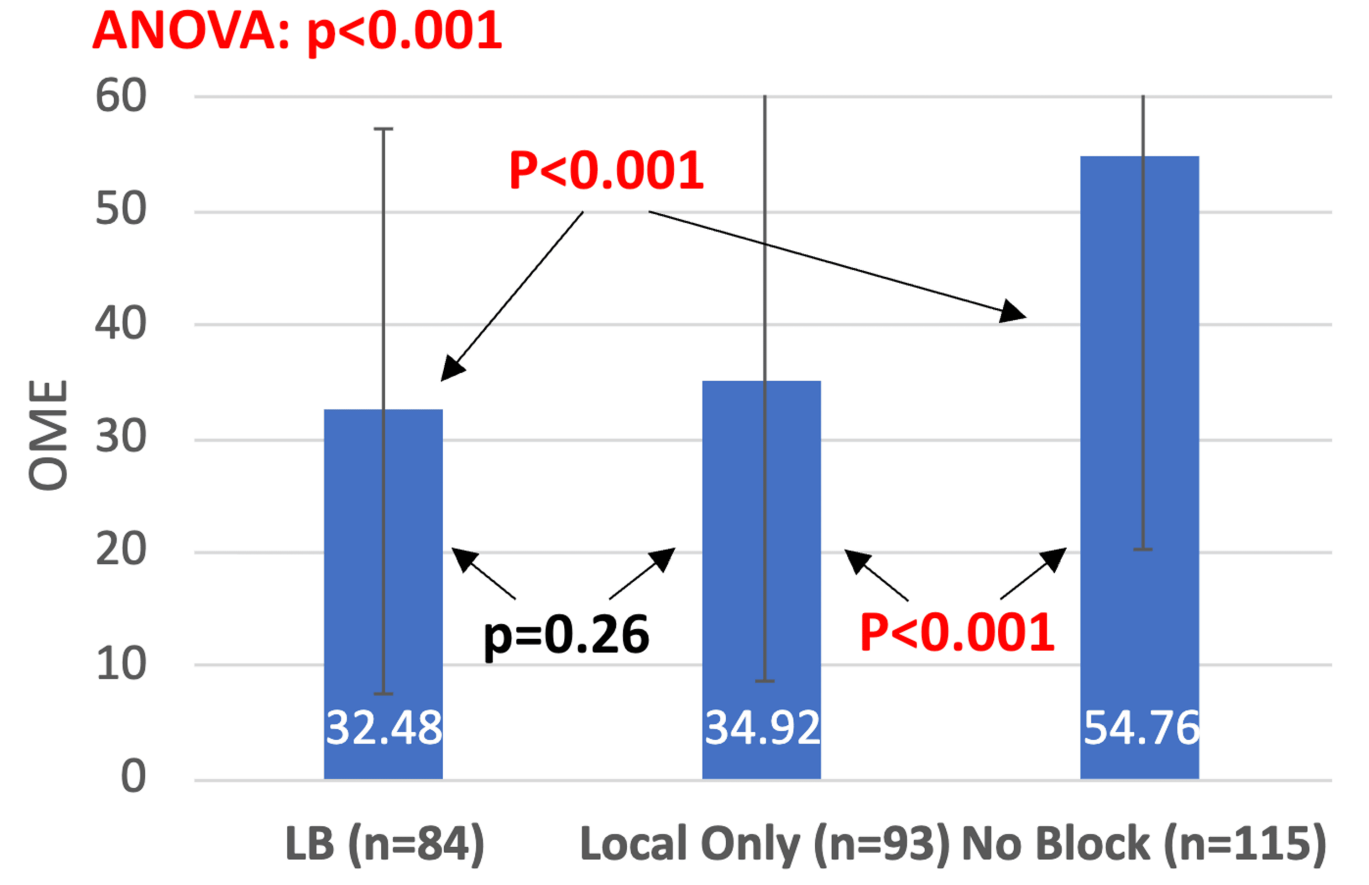
Average OME Use in the First 48 Hours Postoperatively Among Patients in “LB” vs “Local Only” vs “No Block” Groups OME: oral morphine equivalent; LB: liposomal bupivacaine

The primary outcome, average OME use in the first 48 hours postoperatively, was lower in Group 1 compared to Group 3 (32.48 vs. 54.76, p < 0.001). Average OME use in the first 48 hours postoperatively was also lower in Group 2 compared to Group 3 (34.92 vs. 54.76, p < 0.001). However, there was no difference in average OME use in the first 48 hours postoperatively between Groups 1 and 2 (32.48 vs. 34.92, p = 0.26) (Figure 2). 

**Figure 3:**
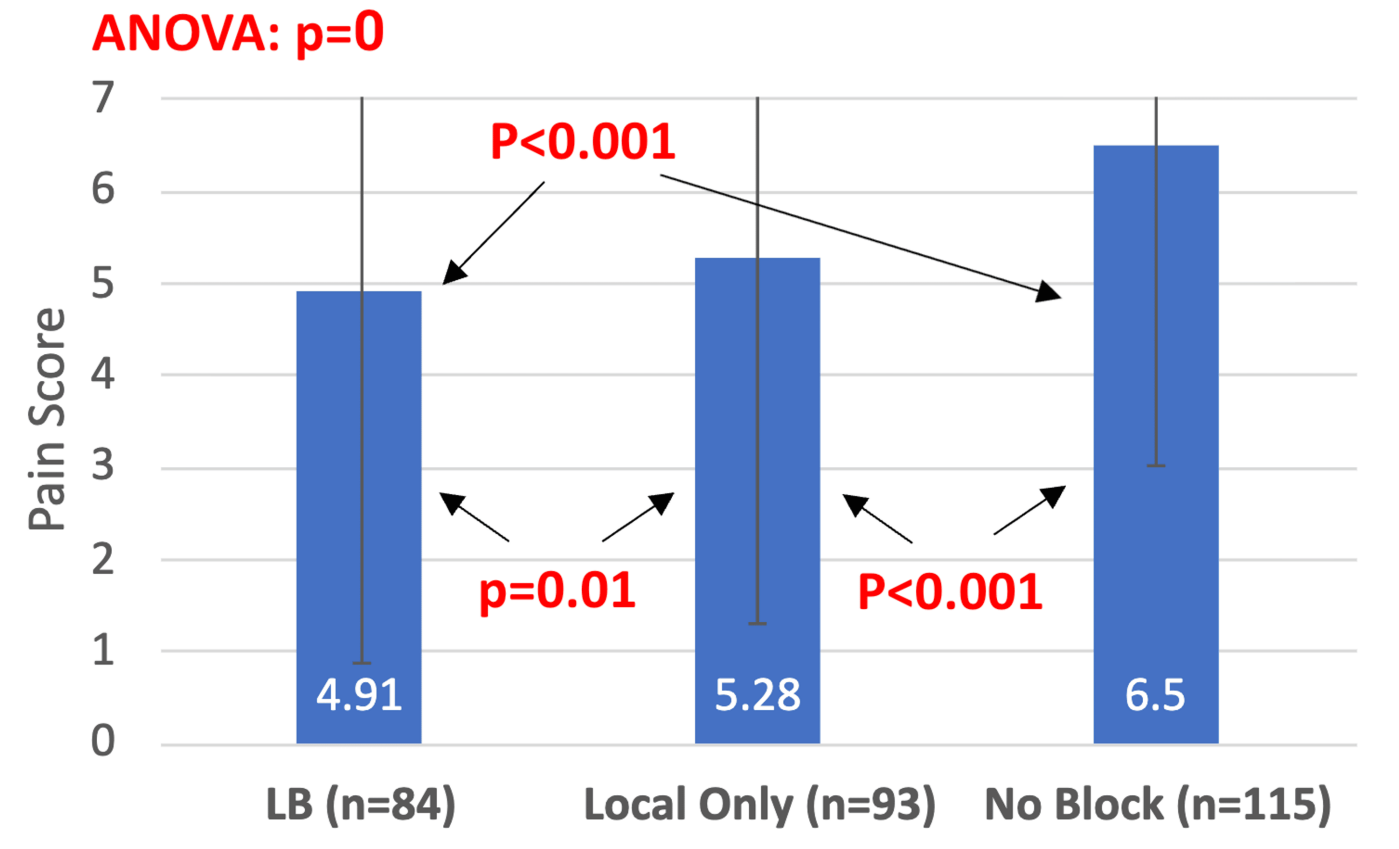
Average Pain Scores in the First 48 Hours Postoperatively Among Patients in “LB” vs “Local Only” vs “No Block” Groups LB: liposomal bupivacaine

The secondary outcome, average pain scores in the first 48 hours postoperatively, were lower in Group 1 compared to both Group 2 (4.91 vs. 5.28, p = 0.01) and Group 3 (4.91 vs. 6.5, p < 0.001). Average pain scores in the first 48 hours postoperatively were also lower in Group 2 compared to Group 3 (5.28 vs. 6.5, p < 0.001) (Figure 3). 

## Discussion

With regards to our secondary outcome, LB did show statistically significant differences in pain scores between the LB block group, local-only block group, and no block group during the first 48 hours after surgery. However, when reviewing the minimal clinically important difference (MCID) of changes in pain intensity and pain scores [36], our pain score values within the numeric rating scale did not change by 2 points (from the LB group of 4.9 to the no block group of 6.5). Thus, clinically, the concept of "much better" improvement with pain [36] from LB was not detected in our study.

Upon reviewing our primary outcome, our study reinforced previous research [37] showing that regional nerve blocks with LB are associated with less OME use in BKA patients in the first 48 hours postoperatively compared to not using a regional nerve block. However, regional nerve blocks with LB were not associated with less OME use in BKA patients in the first 48 hours postoperatively compared to regional nerve blocks with local anesthetic only. This failure to detect a difference in opioid consumption between the two groups is contradictory to other studies supported by Pacira, the manufacturer of LB [38].

For interpretive and supplementary context, these associations raise an ethical question. Furthermore, a point of consideration is that additional local anesthetic cannot be given to patients who have received LB for 96 hours, according to the manufacturer's label for this medication [39]. If the nerve block involving LB did not provide an adequate duration of analgesia, a disadvantage of using LB would be the inability to perform additional nerve blocks postoperatively using local anesthetic for 96 hours.

This conclusion raises an ethical question in the use of LB for BKA patients and makes developing a clear, actionable plan more nuanced. As a physician, LB may be the clear choice if it enables less pain for patients, despite not significantly lowering opioid use. However, given that LB is notably more expensive than local anesthetic [28,33], it is reasonable to question the value of LB use in lower extremity amputation surgery. 

The total hospital cost for a BKA procedure is around $130,000 [40]. Considering one vial of 10 mL of LB (133 mg) costs approximately $315 compared to $4.92-$27 for standard bupivacaine, the cost value of LB may not be significant in the context of the total expenses for BKA surgery. Furthermore, the cost of LB has been reduced below $200 as a result of the NOPAIN Act passed in 2025. Beyond its direct cost, LB may provide downstream economic benefits through a reduction in postoperative opioid consumption and the potential prevention of NPOU. Previous studies have demonstrated that opioid-naive patients who develop NPOU after major surgery incur substantially higher healthcare utilization and significantly greater one-year healthcare costs compared with patients who do not develop NPOU, with increased expenditures observed across inpatient and outpatient settings and among commercial, Medicare, and Medicaid populations [41]. Therefore, even modest reductions in NPOU associated with improved postoperative pain management could produce meaningful cost savings that outweigh the higher upfront cost of LB. It may be argued that the reduced cost of LB and the results of this retrospective study may support the use of LB in BKA surgery [33,40-44].

Limitations of this study include its retrospective, non-randomized design. Confounding by indication is the main concern with this design, where the reason a given patient received their specific treatment may itself be associated with their pain scores or OME. In addition, Table 1 (patient demographics) reveals disparities between the groups, including the no-block group having significantly more elective cases and significant variation among the groups in anxiety/depression, all of which could affect pain and opioid use. Another limitation is the 48-hour window of postoperative data collection, which sheds light on acute pain and early opioid use but does not necessarily allow longer-term conclusions about chronic post-amputation pain, phantom limb pain, or NPOU.

Of note, despite popliteal nerve blocks in adults being approved by the FDA in 2023, the institution where this study was conducted had been administering these blocks off-label since 2019, avoiding temporal confounding within the study.

Future studies should consist of prospective, randomized controlled trials with larger sample sizes for subgroup analyses. Time frames beyond 48 hours postoperatively should also be evaluated for a broader perspective on the beneficial effects of LB on amputation surgery. In addition, a careful cost analysis should be completed so that other hospital systems may make an informed decision on whether to use LB or not.

## Conclusions

LB in regional blocks is associated with lower patient postoperative pain scores following BKA procedures when compared to local-only blocks and to no blocks. However, given its apparently less substantial impact on postoperative opioid use, its clinical utility remains uncertain, especially in the setting of the increased cost of LB. Further research is warranted to draw conclusions via larger, prospective randomized controlled trials with subgroup analyses, to elucidate the effects of LB beyond the 48-hour postoperative period, and to evaluate its cost-benefit in the context of the total cost of BKA surgery.
